# Supplementary material for: DChIPRep, an R/Bioconductor package for differential enrichment analysis in chromatin studies
Source: PeerJ. 2016 Apr 26;4:e1981. doi: 10.7717/peerj.1981 (PMC4860309; doi:10.7717/peerj.1981)
Supplement: Supplemental Information 1 [file peerj-04-1981-s001.html]

Introduction to the DChIPRep package


# Introduction to the DChIPRep package

#### *Bernd Klaus and Christophe Chabbert*

#### *10 March 2016*

# Contents

- 1 Preparations
- 2 Introduction to the DChIPRep package
- 3 Introduction to the pre–processing script
  - 3.1 General principle
  - 3.2 Quick start
  - 3.3 Advanced options
  - 3.4 Example
    - 3.4.1 A note on the count table dimensions
- 4 Importing the data into R
  - 4.1 Importing the output from the Python script
  - 4.2 Importing count matrices
- 5 Importing .bam files directly using `importData_soGGi`
- 6 Perform the tests
- 7 Plots implemented in the package
  - 7.1 Plot the significant positions
  - 7.2 Produce TSS plots
- 8 Session information

# 1 Preparations

We first set global chunk options and load the packages that are necessary to run the vignette.

```
library(DChIPRep)
```

```
## Warning: replacing previous import 'GenomicFeatures::transcriptLengths' by
## 'ensembldb::transcriptLengths' when loading 'ChIPpeakAnno'
```

```
## Warning: replacing previous import 'ggplot2::Position' by 'BiocGenerics::Position' when loading
## 'soGGi'
```

```
library(ggplot2)
library(DESeq2)
```

# 2 Introduction to the DChIPRep package

The package implements the analysis strategy of (Chabbert et al. 2015). Along with an experimental protocol to multiplex ChIP–Seq experiments, (Chabbert et al. 2015) developped a methodology to assess differences between chromatin modification profiles in replicated ChIP–Seq studies that builds on the packages *DESeq2* and *fdrtool*. The package also includes a preprocessing script in Python that allows the user to import sam files into data structures than can be used with the package.

# 3 Introduction to the pre–processing script

Together with the release of the R package *DChIPRep*, we provide a python framework that should allow users to generate count tables. These may then be directly used to evaluate metagene enrichment profiles. This framework is entirely contained in a script that only requires the installation of Python 2.7 and of the packages HTSeq and Numpy, which are free to download. This section describes briefly the various possibilities of analysis offered by this tool together with the various input options and parameters. Additional information may be found directly in the source code or via the usual help framework offered by Python.

The script comes with the package and can be located on your system by the following commands:

```
pythonScriptsDir <- system.file( "exec" , package = "DChIPRep" ) 
pythonScriptsDir
```

```
## [1] "/tmp/RtmpRl2JvH/Rinst702a280eb665/DChIPRep/exec"
```

```
list.files(pythonScriptsDir)
```

```
## [1] "DChIPRep.py"
```

## 3.1 General principle

This script will process alignments of paired-end reads, filter out divergent pairs and low quality alignments and ultimately identify potential PCR duplicates. For the pairs of read retained after filtering, the center of the genomic loci determined by each pair is estimated using mapping coordinates. This center constitutes an approximation of the center of each observed nucleosome. The provided annotation file is then used to estimate the nucleosome counts around the features of interest. These counts are ultimately used to provide a count table in which each row corresponds to one feature and each column to a genomic position around the feature start. This table may then be imported in R using the **importData** function of the *DChIPRep* package.

## 3.2 Quick start

Here we show how to quickly generate a count table from an alignment file. The *DChIPRep.py* script only requires 4 arguments (all other options have a default setting, cf below):

- An alignment file in the SAM format (some HTSeq functions are not currently supported for BAM files) that may be zipped. It is specified by the ‘-i’ option on the command line.
- An annotation file in the gff format (specifications for the gff format may be found here) specified in the ‘-a’ option
- A tabulated file containing the names of each of the considered chromosomes and the size:
- chrI 1304563
- chrII 6536634
- …
- It is crucial to ensure that the chromosome names used in the alignment file and this information file are identical. This information is used by the program to pre–allocate memory to store the counts. A valid path to this text file should be specified under the ‘-g’ option.
- A valid path for an output file specified under the option ‘-o’. A new file will be created automatically - if it already exists, its content will be erased and replaced by the script output.

Here is an example of the command line to be run

```
python DChIPRep.py -i alignment.sam -a my_gff.gff -g chromosome_sizes.txt -o my_count_table.txt
```

## 3.3 Advanced options

In order to provide greater flexibility in data pre-processing, we have added a panel of additional options that may be specified when running the script. The script is executable, With the *–help* option, one can get an overview of the available options. They are given below.

```
usage: DChIPRep.py [-h] -i SAM/BAM -a GFF -g Chromosome Sizes File -o Count Table [-v] [-q TH_QC] [-l TH_LOW] [-L TH_HIGH] [-d TH_PCR] [-f FEATURETYPE]
                   [-w DOWNSTREAM] [-u UPSTREAM]

optional arguments:
  -h, --help            show this help message and exit
  -i SAM/BAM, --input SAM/BAM
                        The alignment file to be used for to generate the count table. The file may be in the sam (zipped or not)format. The extension of
                        the file should contain either the '.sam' indication. Bam files are not supported at the moment due to soome instability in the BAM
                        reader regarding certain aligner formats.
  -a GFF, --annotation GFF
                        The annotation file that will be used to generate the counts. The file should be in the gff format (see
                        https://www.sanger.ac.uk/resources/software/gff/spec.html for details).
  -g Chromosome Sizes File, --genome_details Chromosome Sizes File
                        A tabulated file containing the names and sizes of each chromosome. !!! The the chromosome names should be identical to the ones
                        used to generate the alignment file !!! The file should look like this example (no header): chromI 1304563 chromII 6536634 ...
  -o Count Table, --output_file Count Table
                        The output file where the count table should be stored. If the specified file does not already exist it will be created
                        automatically. Otherwise, it will be overwritten
  -v, --verbose         When specified, the option will result in the printing of informations on the alignments and the filtering steps (number of
                        ambiguous alignments...etc). Default: OFF
  -q TH_QC, --quality_threshold TH_QC
                        The quality threshold below which alignments will be discarded. The alignment quality index typically ranges between 1 and 41.
                        Default: 30.
  -l TH_LOW, --lowest_size TH_LOW
                        The lowest possible size accepted for DNA fragments. Any pair of reads with an insert size below that value will be discarded.
                        Default: 130.
  -L TH_HIGH, --longest_size TH_HIGH
                        The longest possible size accepted for DNA fragments. Any pair of reads with an insert size above this value will be discarded.
                        Default: 180.
  -d TH_PCR, --duplicate_filter TH_PCR
                        The number of estimated PCR duplicates for accepted for a given genomic location. Default: 1.
  -f FEATURETYPE, --feature_type FEATURETYPE
                        The feature types to be used when generating the count table. The feature type will be matched 3rd column of the GFF file. Default:
                        'Transcript
  -w DOWNSTREAM, --downstream_window DOWNSTREAM
                        The window size used to obtain the counts downstream of the TSS. Default: 1000bp
  -u UPSTREAM, --upstream_window UPSTREAM
                        The window size used to obtain the counts upstream of the TSS. Default: 1500bp
```

## 3.4 Example

As an example, let us assume that we want to process an alignment file with the following criteria:

- Remove reads with an alignment quality below 20
- Focus on the pairs with a spanning size ranging between 120 and 160 bp
- Get the counts information around the TSS of the coding sequences (CDS) in a symmetrical window of 500bp
- Only tolerate two copies on the same molecules upstream

The command line would then be:

```
python DChIPRep.py -i alignment.sam -a my_gff.gff 
-g chromosome_sizes.txt -o my_count_table.txt -v -q 20 -l 120 
-L 160 -d 2 -f CDS -u 500 -w 500
```

### 3.4.1 A note on the count table dimensions

The default number of upstream position is 1500bp, the default number of downstream positions is 1000bp. This results in count tables with 2502 columns in total, corresponding to the basepairs up– and downstream as well as one column for  
for the 0 position and one column for the feature IDs.

# 4 Importing the data into R

In what follows, we assume that we count nucleosomes around transcription start sites (TSS), that is the start of transcripts, which is also the default option in the preprocessing script.

## 4.1 Importing the output from the Python script

After the data has been preproccesed, we first need an annotation table for our samples that looks like this:

```
data(exampleSampleTable)
exampleSampleTable
```

```
##                               ChIP                          Input upstream downstream  condition
## 1   BY_conf_K4me3_1__ORF_count.txt   BY_conf_WCE_1__ORF_count.txt     1000       1500   K4me3_WT
## 2   BY_conf_K4me3_2__ORF_count.txt   BY_conf_WCE_2__ORF_count.txt     1000       1500   K4me3_WT
## 3   BY_conf_K4me3_3__ORF_count.txt   BY_conf_WCE_3__ORF_count.txt     1000       1500   K4me3_WT
## 4 SET2_conf_K4me3_1__ORF_count.txt SET2_conf_WCE_1__ORF_count.txt     1000       1500 K4me3_SET2
## 5 SET2_conf_K4me3_2__ORF_count.txt SET2_conf_WCE_2__ORF_count.txt     1000       1500 K4me3_SET2
## 6 SET2_conf_K4me3_3__ORF_count.txt SET2_conf_WCE_3__ORF_count.txt     1000       1500 K4me3_SET2
##       sampleID
## 1   K4me3_WT_1
## 2   K4me3_WT_2
## 3   K4me3_WT_3
## 4 K4me3_SET2_1
## 5 K4me3_SET2_2
## 6 K4me3_SET2_3
```

It gives the names for the input count files in the columns **ChIP** and **Input** respectively and the number of base pairs used for analysis upstream and downstream of the TSS in the columns **upstream** and **downstream**. Note that the number of upstream and downstream positions needs to be the same for all samples.

The input files must be tab separated files with genomic features in the rows and the positions around the TSS in the columns. In addition to the position columns, a column containing a feature ID must be present.

As mentioned above, the tables have upstream + downstream + 2 columns in total, the two extra columns correspond to the center of the profile (e.g. a TSS) and a column containing the feature IDs.

The **sampleID** column contains unique sample IDs.

We can then import the data as follows using the function **importData** which needs the sample annotation table, a `data.frame` and the directory where the raw data files are stored. We use the down–sampled raw data that come with the package to illustrate the function use.

By default the feature ID column is assumed to have the name **name**, however this can specified in the call to **importData** via the **ID** argument.

```
directory <- file.path(system.file("extdata", package="DChIPRep"))
importedData <- importData(exampleSampleTable, directory)
```

The imported data is a DChIPRepResults object that contains the data as a DESeqDataSet. It can be accessed via the function **DESeq2Data**. The DESeqDataSet also contains normalization factors that are equal to the counts from the chromatin inputs, after being multiplied by the coverage ratio between the input and the ChIP data sets.

```
importedData
```

```
## Summary of the DESeqDataSet:
## ============================
## class: DESeqDataSet 
## dim: 2501 6 
## metadata(0):
## assays(2): counts normalizationFactors
## rownames(2501): Pos_-1000 Pos_-999 ... Pos_1499 Pos_1500
## rowData names(0):
## colnames(6): K4me3_WT_1 K4me3_WT_2 ... K4me3_SET2_2 K4me3_SET2_3
## colData names(6): ChIP Input ... condition sampleID
## 
## 
## 
## Results:
## ============================
## No results available yet, call runTesting first
```

```
DESeq2Data(importedData)
```

```
## class: DESeqDataSet 
## dim: 2501 6 
## metadata(0):
## assays(2): counts normalizationFactors
## rownames(2501): Pos_-1000 Pos_-999 ... Pos_1499 Pos_1500
## rowData names(0):
## colnames(6): K4me3_WT_1 K4me3_WT_2 ... K4me3_SET2_2 K4me3_SET2_3
## colData names(6): ChIP Input ... condition sampleID
```

```
head(normalizationFactors(DESeq2Data(importedData)))
```

```
##           K4me3_WT_1 K4me3_WT_2 K4me3_WT_3 K4me3_SET2_1 K4me3_SET2_2 K4me3_SET2_3
## Pos_-1000      4.069     0.8868     2.8974       0.3647         2.58       0.2305
## Pos_-999       5.426     3.1038     1.0865       0.2084         1.29       0.1536
## Pos_-998       1.356     0.8868     0.7244       0.1042         1.29       0.3073
## Pos_-997       8.139     0.4434     1.8109       0.2084         1.72       0.2305
## Pos_-996       2.713     0.8868     3.2596       0.1042         1.29       0.6146
## Pos_-995       2.713     2.2170     2.8974       0.2084         1.72       0.2305
```

## 4.2 Importing count matrices

If your data is already available within R, you can import it directly via **importDataFromMatrices** from an input and a ChIP Matrix. The rows of the matrices should correspond to the positions and the columns to the samples. You furthermore need a sample table as described above, however the columns **Input** and **ChIP** are not needed.

If you have data that is still on the level of the individual features, you can use the helper function **summarizeCountsPerPosition** to summarize the counts for individual features per position.

The package comes with example data sets that correspond to the example sample table shown above.

We first show 10 random rows from the two data matrices and then use the function **importDataFromMatrices** to import them to a **DChIPRepResults** object.

As you can see the rows should be named according to the positions up– and downstream of the TSS that they contain and the columns should be named after the samples.

```
data(exampleInputData)
data(exampleChipData)

exampleSampleTable
```

```
##                               ChIP                          Input upstream downstream  condition
## 1   BY_conf_K4me3_1__ORF_count.txt   BY_conf_WCE_1__ORF_count.txt     1000       1500   K4me3_WT
## 2   BY_conf_K4me3_2__ORF_count.txt   BY_conf_WCE_2__ORF_count.txt     1000       1500   K4me3_WT
## 3   BY_conf_K4me3_3__ORF_count.txt   BY_conf_WCE_3__ORF_count.txt     1000       1500   K4me3_WT
## 4 SET2_conf_K4me3_1__ORF_count.txt SET2_conf_WCE_1__ORF_count.txt     1000       1500 K4me3_SET2
## 5 SET2_conf_K4me3_2__ORF_count.txt SET2_conf_WCE_2__ORF_count.txt     1000       1500 K4me3_SET2
## 6 SET2_conf_K4me3_3__ORF_count.txt SET2_conf_WCE_3__ORF_count.txt     1000       1500 K4me3_SET2
##       sampleID
## 1   K4me3_WT_1
## 2   K4me3_WT_2
## 3   K4me3_WT_3
## 4 K4me3_SET2_1
## 5 K4me3_SET2_2
## 6 K4me3_SET2_3
```

```
exampleInputData[sample(nrow(exampleInputData), 10), ]
```

```
##       K4me3_WT_1 K4me3_WT_2 K4me3_WT_3 K4me3_SET2_1 K4me3_SET2_2 K4me3_SET2_3
## X465         764       1092       1482         1046          982         1023
## X331        1018       1388       1980         1365         1373         1542
## X.841       1442       1987       2948         1698         1749         1878
## X1278       1452       2021       2893         1774         1799         1865
## X.285        939       1296       1911         1137         1092         1153
## X1276       1445       2022       2949         1781         1790         1888
## X.295        859       1262       1785         1032         1102         1117
## X.139        503        827       1102          609          578          590
## X.747       1558       2247       3035         1886         1893         1896
## X867        1913       2669       3769         2140         2105         2286
```

```
exampleChipData[sample(nrow(exampleChipData), 10), ]
```

```
##       K4me3_WT_1 K4me3_WT_2 K4me3_WT_3 K4me3_SET2_1 K4me3_SET2_2 K4me3_SET2_3
## X.172       2653       1345       1502          124          845          176
## X737        5184       2248       2632          224         1829          362
## X1003       4752       2085       2286          235         1755          346
## X1269       3643       1572       1837          232         1487          276
## X.892       4803       2235       2563          223         1804          370
## X357        9146       4146       4731          407         3431          623
## X.389       5214       2491       2906          222         1828          413
## X.973       4046       1756       2047          185         1468          302
## X.450       3380       1596       1884          154         1213          258
## X905        4094       1799       2074          221         1598          304
```

```
imDataFromMatrices <- importDataFromMatrices(inputData = exampleInputData, 
                                              chipData = exampleChipData, 
                                              sampleTable = exampleSampleTable)
```

The imported data is again a **DChIPRepResults** object that contains the data as a **DESeqDataSet**.

# 5 Importing .bam files directly using `importData_soGGi`

It is also possible to use the function `importData_soGGi`, which is based on the function `regionPlot` from the package *soGGi* to import data from .bam files directly.

It will return a matrix with one column per .bam file and the respective counts per postion in the rows.

In the example below, we use a subsampled .bam file (0.1 % of the reads) from the Galonska et. al. WCE (whole cell extract) H3Kme3 data and associated TSS near identified peaks. For additional details on the data, see the help pages on `input_galonska` and `TSS_galonska`. The code below is not evaluated, since it takes some time to compute.

```
data(sample_table_galonska)
data(TSS_galonska)

bam_dir <- file.path(system.file("extdata", package="DChIPRep"))
wce_bam <- "subsampled_0001_pc_SRR2144628_WCE_bowtie2_mapped-only_XS-filt_no-dups.bam"
mat_wce <- importData_soGGi(bam_paths = file.path(bam_dir, wce_bam),
                         TSS = TSS_galonska,
                         fragment_lengths = sample_table_galonska$input_fragment_length[1],
                         sample_ids =  sample_table_galonska$input[1],
                      paired = FALSE,
                        removeDup=FALSE
)

head(mat_wce)
```

# 6 Perform the tests

After the data import, we are ready to perform the tests for differential enrichment. The tests are performed position–wise and wrap *DESeq2* and *fdrtool*. Briefly the DChIPRep testing workflow is as follows:

1. The function **estimateDispersions** from *DESeq2* is called and the dispersions are estimated.
2. Then the position–wise tests to compare the experimental conditions are performed. A minimum fold change is used for the null hypothesis, the default value used is 0.05 on a log2 scale.

A possible strategy to infer this threshold from the data is to look a the average fold–change between technical replicates.

3. The p–values are then passed to *fdrtool* and the local FDR values are computed.

```
imDataFromMatrices  <- runTesting(imDataFromMatrices, plotFDR = TRUE)
```

```
## Step 1... determine cutoff point
## Step 2... estimate parameters of null distribution and eta0
## Step 3... compute p-values and estimate empirical PDF/CDF
## Step 4... compute q-values and local fdr
## Step 5... prepare for plotting
```

The results can now be accessed via the function **resultsDChIPRep**.

```
res <- resultsDChIPRep(imDataFromMatrices)
head(res)
```

```
##           baseMean log2FoldChange   lfcSE     stat pvalue padj lfdr
## Pos_-1000   1013.4       -0.03018 0.05040  0.00000 1.0000 1.00    1
## Pos_-999     978.5       -0.03842 0.05181  0.00000 1.0000 1.00    1
## Pos_-998     996.3        0.05452 0.05073  0.08903 0.9291 1.00    1
## Pos_-997    1056.4       -0.04688 0.04821  0.00000 1.0000 1.00    1
## Pos_-996    1011.3       -0.11971 0.04906 -1.42095 0.1553 0.74    1
## Pos_-995    1005.2       -0.06855 0.05002 -0.37082 0.7108 1.00    1
```

```
table( res$lfdr < 0.2)
```

```
## 
## FALSE  TRUE 
##  2407    94
```

At an lfdr of 0.2 we identify 94 significant positions.

# 7 Plots implemented in the package

## 7.1 Plot the significant positions

We can first of all plot the TSS profiles by coloring the individual points by significance.

Points corresponding to significant positions are colored black in both of the conditions. The replicate–samples are sumarized by using a positionwise robust Huber estimator for the mean (Hampel, Hennig, and Ronchetti 2011).

The function returns the plot as a ggplot2 object that can be modified afterwards.

```
sigPlot <- plotSignificance(imDataFromMatrices)
sigPlot
```

This plot is similar to Figure S17B of (Chabbert et al. 2015). We see an enrichment for significant position near the end of the downstream window considered.

## 7.2 Produce TSS plots

We can produce the typical, smoothed plots of the TSS profiles as well. Here we use again the smoothed Huber estimator for the mean to compute a summary per experimental group.

```
profilePlot <- plotProfiles(imDataFromMatrices)
profilePlot
```

This plot is similar to Figure 5B of (Chabbert et al. 2015).

# 8 Session information

```
sessionInfo()
```

```
## R Under development (unstable) (2016-03-07 r70280)
## Platform: x86_64-pc-linux-gnu (64-bit)
## Running under: CentOS release 6.5 (Final)
## 
## locale:
##  [1] LC_CTYPE=en_US.UTF-8       LC_NUMERIC=C               LC_TIME=en_US.UTF-8       
##  [4] LC_COLLATE=en_US.UTF-8     LC_MONETARY=en_US.UTF-8    LC_MESSAGES=en_US.UTF-8   
##  [7] LC_PAPER=en_US.UTF-8       LC_NAME=C                  LC_ADDRESS=C              
## [10] LC_TELEPHONE=C             LC_MEASUREMENT=en_US.UTF-8 LC_IDENTIFICATION=C       
## 
## attached base packages:
## [1] parallel  stats4    stats     graphics  grDevices utils     datasets  methods   base     
## 
## other attached packages:
##  [1] ggplot2_2.1.0               DChIPRep_1.1.7              RSQLite_1.0.0              
##  [4] DBI_0.3.1                   DESeq2_1.11.23              SummarizedExperiment_1.1.21
##  [7] Biobase_2.31.3              GenomicRanges_1.23.24       GenomeInfoDb_1.7.6         
## [10] IRanges_2.5.39              S4Vectors_0.9.41            BiocGenerics_0.17.3        
## [13] rmarkdown_0.9.5             knitr_1.12.3                BiocStyle_1.9.5            
## 
## loaded via a namespace (and not attached):
##  [1] nlme_3.1-125                 bitops_1.0-6                 matrixStats_0.50.1          
##  [4] smoothmest_0.1-2             RColorBrewer_1.1-2           httr_1.1.0                  
##  [7] tools_3.3.0                  R6_2.1.2                     rpart_4.1-10                
## [10] mgcv_1.8-12                  lazyeval_0.1.10              Hmisc_3.17-2                
## [13] colorspace_1.2-6             ade4_1.7-4                   nnet_7.3-12                 
## [16] gridExtra_2.2.1              preprocessCore_1.33.0        VennDiagram_1.6.16          
## [19] fdrtool_1.2.15               graph_1.49.1                 formatR_1.3                 
## [22] labeling_0.3                 rtracklayer_1.31.7           scales_0.4.0                
## [25] genefilter_1.53.1            RBGL_1.47.0                  stringr_1.0.0               
## [28] digest_0.6.9                 Rsamtools_1.23.3             foreign_0.8-66              
## [31] XVector_0.11.7               htmltools_0.3                ensembldb_1.3.17            
## [34] limma_3.27.13                BSgenome_1.39.4              regioneR_1.3.2              
## [37] BiocInstaller_1.21.3         shiny_0.13.1                 hwriter_1.3.2               
## [40] BiocParallel_1.5.19          dplyr_0.4.3                  acepack_1.3-3.3             
## [43] RCurl_1.95-4.8               magrittr_1.5                 GO.db_3.2.2                 
## [46] Formula_1.2-1                Matrix_1.2-4                 futile.logger_1.4.1         
## [49] Rcpp_0.12.3                  munsell_0.4.3                stringi_1.0-1               
## [52] yaml_2.1.13                  MASS_7.3-45                  zlibbioc_1.17.0             
## [55] plyr_1.8.3                   AnnotationHub_2.3.14         grid_3.3.0                  
## [58] lattice_0.20-33              Biostrings_2.39.12           splines_3.3.0               
## [61] multtest_2.27.0              GenomicFeatures_1.23.25      annotate_1.49.1             
## [64] locfit_1.5-9.1               soGGi_1.3.2                  seqinr_3.1-3                
## [67] codetools_0.2-14             geneplotter_1.49.0           reshape2_1.4.1              
## [70] biomaRt_2.27.2               futile.options_1.0.0         XML_3.98-1.4                
## [73] ShortRead_1.29.2             evaluate_0.8.3               latticeExtra_0.6-28         
## [76] lambda.r_1.1.7               idr_1.2                      httpuv_1.3.3                
## [79] tidyr_0.4.1                  gtable_0.2.0                 purrr_0.2.1                 
## [82] assertthat_0.1               chipseq_1.21.2               mime_0.4                    
## [85] xtable_1.8-2                 survival_2.38-3              ChIPpeakAnno_3.5.7          
## [88] GenomicAlignments_1.7.20     AnnotationDbi_1.33.7         memoise_1.0.0               
## [91] cluster_2.0.3                interactiveDisplayBase_1.9.0
```

# References

Chabbert, C. D., S. H. Adjalley, B. Klaus, E. S. Fritsch, I. Gupta, V. Pelechano, and L. M. Steinmetz. 2015. “A High-Throughput ChIP-Seq for Large-Scale Chromatin Studies.” *Molecular Systems Biology* 11 (1). EMBO: 777–77. doi:10.15252/msb.20145776.

Hampel, Frank, Christian Hennig, and Elvezio Ronchetti. 2011. “A Smoothing Principle for the Huber and Other Location M-Estimators.” *Computational Statistics & Data Analysis* 55 (1). Elsevier BV: 324–37. doi:10.1016/j.csda.2010.05.001.
