# Supplementary material for: DChIPRep, an R/Bioconductor package for differential enrichment analysis in chromatin studies
Source: PeerJ. 2016 Apr 26;4:e1981. doi: 10.7717/peerj.1981 (PMC4860309; doi:10.7717/peerj.1981)
Supplement: Supplemental Information 2 [file peerj-04-1981-s002.zip › ReproduceFiguresDChIPRepPaper.html]

Analysis code for the article DChIPRep, an R/Bioconductor package for differential enrichment analysis in chromatin studies


# Analysis code for the article “DChIPRep, an R/Bioconductor package for differential enrichment analysis in chromatin studies”

#### *Bernd Klaus and Christophe Chabbert*

#### *5 April 2016*

# Contents

- 1 Required packages and other preparations
- 2 DChIPRep case study – Figure 1
- 3 A csaw/edgeR pipeline – Figure 2
  - 3.1 Overlap between DESeq2 and edgeR
- 4 Galonka case study – Figure 3
- 5 Output of sessionInfo()

# 1 Required packages and other preparations

```
library(knitr)
library(DChIPRep)
library(purrr)
library(BiocStyle)
library(ggplot2)
library(plyr)
library(dplyr)
library(RColorBrewer)
library(stringr)
library(pheatmap)
library(openxlsx)
library(devtools)
library(biomaRt)
library(tidyr)
library(csaw)
library(DESeq2)
library(edgeR)
library(fdrtool)
library(reshape2)
library(smoothmest)
library(gplots)
library(cowplot)
```

# 2 DChIPRep case study – Figure 1

We first load the data from a pre–saved `DChIPRepResults` object. We also import the sample annotation table than contains information on the samples used.

```
load("importedData.RData")

sampleTable_K4me2 <- read.csv("sampleTable_K4me2.csv")
```

```
kable(sampleTable_K4me2, format = "html")
```

| ChIP | Input | upstream | downstream | condition | sampleID |
| --- | --- | --- | --- | --- | --- |
| BY\_conf\_K4me2\_1\_\_ORF\_count.txt | BY\_conf\_WCE\_1\_\_ORF\_count.txt | 1000 | 1500 | WT | BY\_K4me2\_1 |
| BY\_conf\_K4me2\_2\_\_ORF\_count.txt | BY\_conf\_WCE\_2\_\_ORF\_count.txt | 1000 | 1500 | WT | BY\_K4me2\_2 |
| BY\_conf\_K4me2\_3\_\_ORF\_count.txt | BY\_conf\_WCE\_3\_\_ORF\_count.txt | 1000 | 1500 | WT | BY\_K4me2\_3 |
| SET2\_conf\_K4me2\_1\_\_ORF\_count.txt | SET2\_conf\_WCE\_1\_\_ORF\_count.txt | 1000 | 1500 | mutant | BY\_K4me2\_1 |
| SET2\_conf\_K4me2\_2\_\_ORF\_count.txt | SET2\_conf\_WCE\_2\_\_ORF\_count.txt | 1000 | 1500 | mutant | BY\_K4me2\_2 |
| SET2\_conf\_K4me2\_3\_\_ORF\_count.txt | SET2\_conf\_WCE\_3\_\_ORF\_count.txt | 1000 | 1500 | mutant | BY\_K4me2\_3 |

Then we can perfom the testing and produce the TSS plot in Figure 1.

```
testDChIPRep <- runTesting(importedData, plotFDR = FALSE)
```

```
   gene-wise dispersion estimates
   mean-dispersion relationship
   final dispersion estimates
```

```
   Step 1... determine cutoff point
   Step 2... estimate parameters of null distribution and eta0
   Step 3... compute p-values and estimate empirical PDF/CDF
   Step 4... compute q-values and local fdr
```

```
plotSignificance(testDChIPRep)
```

```
### Get the number of bases with significant changes
resDChIPRep <- resultsDChIPRep(testDChIPRep)

table(resDChIPRep$lfdr < 0.2)
```

```
   FALSE  TRUE 
    1571   930
```

```
lfdrDChIPRep <- resDChIPRep$lfdr[1001:2501]
sum(lfdrDChIPRep < 0.2)
```

```
   [1] 906
```

# 3 A csaw/edgeR pipeline – Figure 2

We use edgeR based testing instead of DESeq2 for the testing. For this we extract the raw counts from the data and then run the tesing pipeline just like *csaw* does it, using the quasi–likelihood based testing pipeline implemented in *edgeR*.

Not that it is not possible to define a log–fold–change cutoff for testing in *edgeR*, so we use a post–hoc cutoff to determine significance.

We then produce a plot similar to Figure 1.

```
# extract counts
ccc <- counts(DESeq2Data(importedData))


condition <- colData(DESeq2Data(importedData))$condition
y <- DGEList(ccc, 
             group = condition)

nf <- normalizationFactors(DESeq2Data(importedData))

y$offset <- log(nf)

design <- model.matrix(~ 0 +  condition)
colnames(design) <- levels(condition)
y <- estimateDisp(y, design = design)
plotBCV(y)
```

```
fit <- glmQLFit(y, design, robust=TRUE)
plotQLDisp(fit)
```

```
contrast <- makeContrasts(WT-mutant, levels=design)
res <- glmQLFTest(fit, contrast=contrast)

resEdgeR <- topTags(sort.by = "none", res, n=Inf)$table

FDRedgeR <- fdrtool(resEdgeR$PValue, statistic = "pvalue", plot = FALSE)
```

```
   Step 1... determine cutoff point
   Step 2... estimate parameters of null distribution and eta0
   Step 3... compute p-values and estimate empirical PDF/CDF
   Step 4... compute q-values and local fdr
```

```
resEdgeR$ldfr <- FDRedgeR$lfdr

table(resEdgeR$ldfr < 0.2)
```

```
   FALSE  TRUE 
    1162  1339
```

```
lfdrEdgeR <- FDRedgeR$lfdr[1001:2501]
sum(lfdrEdgeR < 0.2)
```

```
   [1] 1171
```

```
countsLog2 <- as.data.frame(log2(counts(DESeq2Data(importedData), norm = TRUE)))


sampleTable <- colData(DESeq2Data(importedData))

pos <-   seq(from =-unique(sampleTable$upstream)
                            , to = unique(sampleTable$downstream), by = 1)

suppressMessages(c.ggplot2 <- data.frame(pos = pos,
                        melt(countsLog2,
                                       variable.name= "sample",
                                       value.name= "PosSignal")))

### get mean per group for ratio plots
m.f <- function(x) { smhuber(x)$mu }
m.per.group <- t(aggregate.data.frame(t(countsLog2),
                                      by = list(sampleTable$condition), FUN = m.f )[,-1])
colnames(m.per.group) <-  levels(sampleTable$condition)


# introduce post hoc l2fc cutoff
idxSig <- resEdgeR$ldfr < 0.2 & (abs(resEdgeR$logFC / log(2)) > 0.05)

#idxSig <- resEdgeR$FDR < 0.05 & (abs(resEdgeR$logFC / log(2)) > 0.05)

m.per.group <- as.data.frame(m.per.group)
m.per.group$pos <- pos
m.per.group$significant <- idxSig

dataGG <- gather(m.per.group,
       key = "experimental_Group_and_significance",
       value = "mean_log2_counts", 1:2)

dataGG$experimental_Group_and_significance <- ifelse(dataGG$significant,
                                                      "significant",
                                                     as.character(dataGG$experimental_Group_and_significance))

dataGG$experimental_Group_and_significance  <- mapvalues(dataGG$experimental_Group_and_significance,
          from = c("mutant", "WT", "significant"), to = c("Mutant", "Wild type", "Significant Difference"))

dataGG$experimental_Group_and_significance <- factor(dataGG$experimental_Group_and_significance,
                                                     levels = c("Mutant", "Wild type", "Significant Difference"))

pl <- (ggplot(data = dataGG, 
              aes_string(x = "pos",
                         y = "mean_log2_counts",
                         color = "experimental_Group_and_significance")) +
      geom_point() +
      labs(x ="Distance from TSS (bp)",
           y="Normalized counts (log2)") +
      scale_color_manual(values = c( "#d95f02", "#7570b3", "black"), name = "") +
      theme(panel.background = element_blank(), panel.grid.minor=element_blank(),
      axis.line = element_line(colour = "black", size = 0.5)))


pl <- (pl + theme( axis.ticks.length = unit(10, "points"),
             axis.line = element_line(size = 1),
              axis.title = element_text(size = 16, face = "bold"),
             axis.text = element_text(size = 12, face = "bold"),
             plot.title = element_text(size = 16, face = "bold"),
             legend.position = c(0.4, .7),
             legend.key = element_rect(fill = "white"),
             legend.text = element_text(size = 16, face = "bold")
            )
+ ggtitle("Significantly enriched regions based on csaw/edgeR"))


# add previous results as an additional panel

DChIPRepPlot <- (plotSignificance(testDChIPRep) 
            + theme( axis.ticks.length = unit(10, "points"),
             axis.line = element_line(size = 1),
              axis.title = element_text(size = 16, face = "bold"),
             axis.text = element_text(size = 12, face = "bold"),
             plot.title = element_text(size = 16, face = "bold"),
             legend.position = c(0.4, .7),
             legend.key = element_rect(fill = "white"),
             legend.text = element_text(size = 16, face = "bold"),
             panel.background = element_blank(), panel.grid.minor=element_blank(),
            axis.line = element_line(colour = "black", size = 0.5)
            )
            + labs(x ="Distance from TSS (bp)",
           y="Normalized counts (log2)")
           + guides(color = guide_legend(title = NULL))
           +  ggtitle("Significantly enriched regions based on DChIPRep")
           + scale_color_manual(values = c("#3366CC", "#e41a1c", "black"), 
                                labels = c("Mutant", "Wild type", "Significant Difference")))
```

```
   Scale for 'colour' is already present. Adding another scale for 'colour',
   which will replace the existing scale.
```

```
#svg(filename = 'K4me2_significance_edgeR.svg',height = 6, width = 15)
plot_grid(DChIPRepPlot, pl, labels = c("A", "B"), ncol = 2, align = "h")
```

```
#dev.off()
```

## 3.1 Overlap between DESeq2 and edgeR

Her we look at the overlap of significant positions between edgeR and DESeq based testing. THis shows that all DESeq (A) positions are also called by edgeR (B).

```
positions <- rownames(DESeq2Data(testDChIPRep))[1001:2501]

DESeq <- positions[lfdrDChIPRep < 0.2]
edgeR <- positions[lfdrEdgeR < 0.2]

ll <- list(DESeq, edgeR )

venn(ll)
```

```
#venn.diagram(ll, filename = "test.tiff", category.names = c("A", "B"))

#how many positions up to 250 bp downstream are shared?
table(as.integer(str_replace(intersect(DESeq[1:500],
                                       edgeR[1:500]), "Pos_", "")) < 250)
```

```
   FALSE  TRUE 
     361     5
```

```
# only 1% (5)!
```

# 4 Galonka case study – Figure 3

We first load the data from as count matrices for input and ChiP–Seq as well as sample annotation table and then import the data using the `importDataFromMatrices` function.

```
load("galonskaData.rda")

sample_table_galonska
```

```
                  sampleID fragment_length input_fragment_length   input upstream
   1  mES_H3K4me3_Serum_r1             265                   150 mES_WCE     1000
   2  mES_H3K4me3_Serum_r2             260                   150 mES_WCE     1000
   3  ES_H3K4me3_24h_2i_r1             260                   150 mES_WCE     1000
   4 mES_H3K4me3_24h_2i_r2             260                   150 mES_WCE     1000
     downstream condition
   1       1500     Serum
   2       1500     Serum
   3       1500    24h_2i
   4       1500    24h_2i
```

We now run the testing and produce the figure.

```
DChIPRep_galonska <- importDataFromMatrices(input_galonska, 
                                            chip_galonska, 
                                            sample_table_galonska)

plotProfiles(DChIPRep_galonska)
```

```
DChIPRep_galonska  <- runTesting(DChIPRep_galonska, lfcThreshold = 0.00, plotFDR = TRUE)
```

```
   gene-wise dispersion estimates
```

```
   mean-dispersion relationship
```

```
   final dispersion estimates
```

```
   Step 1... determine cutoff point
   Step 2... estimate parameters of null distribution and eta0
   Step 3... compute p-values and estimate empirical PDF/CDF
   Step 4... compute q-values and local fdr
   Step 5... prepare for plotting
```

```
res_galonska <- resultsDChIPRep(DChIPRep_galonska)

table(res_galonska$lfdr < 0.3)
```

```
   FALSE  TRUE 
     641  1860
```

```
pl_galonksa <- plotSignificance(DChIPRep_galonska, lfdrThresh = 0.3)


pl_galonksa <- (pl_galonksa  + theme( axis.ticks.length = unit(10, "points"),
             axis.line = element_line(size = 1),
              axis.title = element_text(size = 16, face = "bold"),
             axis.text = element_text(size = 12, face = "bold"),
             plot.title = element_text(size = 16, face = "bold"),
             legend.position = c(0.55, .42),
             legend.key = element_rect(fill = "white"),
             legend.text = element_text(size = 16, face = "bold"),
             panel.background = element_blank(), panel.grid.minor=element_blank(),
            axis.line = element_line(colour = "black", size = 0.5))
          
            + scale_color_manual(values = c( "#36B522", "#D06EE6", "grey40"), guide = 
                                   guide_legend(title = NULL)) 
            + labs(y="Normalized counts (log2)")
            + ggtitle("Significantly enriched regions for the H3K4me3 data from Galonska et. al."))
```

```
   Scale for 'colour' is already present. Adding another scale for 'colour',
   which will replace the existing scale.
```

```
#svg(filename = 'H3K4me3_golonska.svg',height = 6, width = 15)
pl_galonksa
```

```
#dev.off()
```

# 5 Output of sessionInfo()

```
sessionInfo()
```

```
   R version 3.2.2 (2015-08-14)
   Platform: x86_64-pc-linux-gnu (64-bit)
   Running under: CentOS release 6.5 (Final)
   
   locale:
    [1] LC_CTYPE=en_US.UTF-8       LC_NUMERIC=C              
    [3] LC_TIME=en_US.UTF-8        LC_COLLATE=en_US.UTF-8    
    [5] LC_MONETARY=en_US.UTF-8    LC_MESSAGES=en_US.UTF-8   
    [7] LC_PAPER=en_US.UTF-8       LC_NAME=C                 
    [9] LC_ADDRESS=C               LC_TELEPHONE=C            
   [11] LC_MEASUREMENT=en_US.UTF-8 LC_IDENTIFICATION=C       
   
   attached base packages:
    [1] grid      stats4    parallel  stats     graphics  grDevices utils    
    [8] datasets  methods   base     
   
   other attached packages:
    [1] cowplot_0.6.1                            
    [2] TxDb.Dmelanogaster.UCSC.dm3.ensGene_3.2.2
    [3] pasilla_0.10.0                           
    [4] BiocInstaller_1.20.1                     
    [5] magrittr_1.5                             
    [6] multtest_2.26.0                          
    [7] TeachingDemos_2.10                       
    [8] gplots_2.17.0                            
    [9] MSnbase_1.18.1                           
   [10] ProtGenerics_1.2.1                       
   [11] BiocParallel_1.4.3                       
   [12] mzR_2.4.1                                
   [13] GenomicAlignments_1.6.3                  
   [14] Rsamtools_1.22.0                         
   [15] soGGi_1.2.1                              
   [16] rtracklayer_1.30.3                       
   [17] purrr_0.2.1                              
   [18] readr_0.2.2                              
   [19] ChIPpeakAnno_3.4.6                       
   [20] RSQLite_1.0.0                            
   [21] DBI_0.3.1                                
   [22] VennDiagram_1.6.16                       
   [23] futile.logger_1.4.1                      
   [24] Biostrings_2.38.4                        
   [25] XVector_0.10.0                           
   [26] TxDb.Mmusculus.UCSC.mm9.knownGene_3.2.2  
   [27] GenomicFeatures_1.22.13                  
   [28] AnnotationDbi_1.32.3                     
   [29] smoothmest_0.1-2                         
   [30] MASS_7.3-45                              
   [31] reshape2_1.4.1                           
   [32] fdrtool_1.2.15                           
   [33] xtable_1.8-2                             
   [34] edgeR_3.12.0                             
   [35] limma_3.26.9                             
   [36] DESeq2_1.10.1                            
   [37] RcppArmadillo_0.6.600.4.0                
   [38] Rcpp_0.12.3                              
   [39] csaw_1.4.1                               
   [40] SummarizedExperiment_1.0.2               
   [41] Biobase_2.30.0                           
   [42] GenomicRanges_1.22.4                     
   [43] GenomeInfoDb_1.6.3                       
   [44] IRanges_2.4.8                            
   [45] S4Vectors_0.8.11                         
   [46] BiocGenerics_0.16.1                      
   [47] tidyr_0.4.1                              
   [48] biomaRt_2.26.1                           
   [49] devtools_1.10.0                          
   [50] openxlsx_3.0.0                           
   [51] pheatmap_1.0.8                           
   [52] stringr_1.0.0                            
   [53] RColorBrewer_1.1-2                       
   [54] ggplot2_2.1.0                            
   [55] DChIPRep_1.0.4                           
   [56] dplyr_0.4.3                              
   [57] plyr_1.8.3                               
   [58] knitr_1.12.3                             
   [59] BiocStyle_1.8.0                          
   
   loaded via a namespace (and not attached):
    [1] colorspace_1.2-6             hwriter_1.3.2               
    [3] affyio_1.40.0                interactiveDisplayBase_1.8.0
    [5] codetools_0.2-14             splines_3.2.2               
    [7] doParallel_1.0.10            impute_1.44.0               
    [9] geneplotter_1.48.0           Formula_1.2-1               
   [11] annotate_1.48.0              vsn_3.38.0                  
   [13] cluster_2.0.3                GO.db_3.2.2                 
   [15] graph_1.48.0                 shiny_0.13.1                
   [17] httr_1.1.0                   lazyeval_0.1.10             
   [19] assertthat_0.1               formatR_1.3                 
   [21] acepack_1.3-3.3              htmltools_0.3.5             
   [23] tools_3.2.2                  affy_1.48.0                 
   [25] gtable_0.2.0                 MALDIquant_1.14             
   [27] ShortRead_1.28.0             gdata_2.17.0                
   [29] preprocessCore_1.32.0        iterators_1.0.8             
   [31] mime_0.4                     ensembldb_1.2.2             
   [33] gtools_3.5.0                 statmod_1.4.24              
   [35] XML_3.98-1.4                 AnnotationHub_2.2.5         
   [37] zlibbioc_1.16.0              scales_0.4.0                
   [39] BSgenome_1.38.0              pcaMethods_1.60.0           
   [41] RBGL_1.46.0                  lambda.r_1.1.7              
   [43] yaml_2.1.13                  memoise_1.0.0               
   [45] gridExtra_2.2.1              rpart_4.1-10                
   [47] latticeExtra_0.6-28          stringi_1.0-1               
   [49] genefilter_1.52.1            foreach_1.4.3               
   [51] caTools_1.17.1               chipseq_1.20.0              
   [53] bitops_1.0-6                 matrixStats_0.50.1          
   [55] mzID_1.8.0                   evaluate_0.8.3              
   [57] lattice_0.20-33              labeling_0.3                
   [59] R6_2.1.2                     Hmisc_3.17-2                
   [61] foreign_0.8-66               survival_2.38-3             
   [63] RCurl_1.95-4.8               nnet_7.3-12                 
   [65] futile.options_1.0.0         KernSmooth_2.23-15          
   [67] rmarkdown_0.9.5              locfit_1.5-9.1              
   [69] digest_0.6.9                 httpuv_1.3.3                
   [71] regioneR_1.2.3               munsell_0.4.3
```
