# Supplementary material for: DChIPRep, an R/Bioconductor package for differential enrichment analysis in chromatin studies
Source: PeerJ. 2016 Apr 26;4:e1981. doi: 10.7717/peerj.1981 (PMC4860309; doi:10.7717/peerj.1981)
Supplement: Supplemental Information 3 [file peerj-04-1981-s003.html]

Preprocessing of the golonska H3K4me3 histone modification data


# Preprocessing of the golonska H3K4me3 histone modification data

#### *Bernd Klaus and Christophe Chabbert*

#### *11 March 2016*

# Contents

- 1 Required packages and other preparations
- 2 About this document
- 3 Import peak lists from paper and annotate nearest TSS
- 4 Get the TSS
- 5 Get the count tables for the samples
- 6 Output of sessionInfo()

# 1 Required packages and other preparations

```
library(knitr)
library(BiocStyle)
library(DChIPRep)
library(ggplot2)
library(dplyr)
library(tidyr)
library(TxDb.Mmusculus.UCSC.mm9.knownGene)
library(ChIPpeakAnno) 
library(readr)
library(stringr)
library(purrr)
library(rtracklayer)
library(stringr)
library(soGGi)
library(GenomicRanges)
library(GenomicAlignments)


mus_genes  <- annoGR(TxDb.Mmusculus.UCSC.mm9.knownGene, feature="gene")
info(mus_genes)
```

```
   annoGR object;
   # source:  TxDb.Mmusculus.UCSC.mm9.knownGene 
   # create at:  Tue Mar 08 12:00:00 AM 2016 UTC 
   # feature:  gene 
   # Db type: TxDb
   # Supporting package: GenomicFeatures
   # Data source: UCSC
   # Genome: mm9
   # Organism: Mus musculus
   # Taxonomy ID: 10090
   # UCSC Table: knownGene
   # Resource URL: http://genome.ucsc.edu/
   # Type of Gene ID: Entrez Gene ID
   # Full dataset: yes
   # miRBase build ID: NA
   # transcript_nrow: 55419
   # exon_nrow: 246570
   # cds_nrow: 213117
   # Db created by: GenomicFeatures package from Bioconductor
   # Creation time: 2015-10-07 18:13:02 +0000 (Wed, 07 Oct 2015)
   # GenomicFeatures version at creation time: 1.21.30
   # RSQLite version at creation time: 1.0.0
   # DBSCHEMAVERSION: 1.1
```

```
mus_genes
```

```
   annoGR object with 21677 ranges and 0 metadata columns:
               seqnames                 ranges strand
                  <Rle>              <IRanges>  <Rle>
     100009600     chr9 [ 20866837,  20872369]      -
     100009609     chr7 [ 92088679,  92112519]      -
     100009614    chr10 [ 77174202,  77174754]      +
        100012     chr4 [143747459, 143752554]      -
        100017     chr4 [134301327, 134323919]      -
           ...      ...                    ...    ...
         99889     chr3 [ 84299986,  85691440]      -
         99890     chr3 [110049028, 110053916]      -
         99899     chr3 [151393887, 151412923]      -
         99929     chr3 [ 65332369,  65359440]      +
         99982     chr4 [136106448, 136158638]      -
     -------
     seqinfo: 35 sequences (1 circular) from mm9 genome
```

```
bam_dir <- file.path("/g/huber/users/klaus/Data/ChiP-Seq-MouseESCs_2i/galaxy_out/data/bam/mapped-only_XS-filt_nodups")

sample_table <- read_csv(file.path("/g/huber/users/klaus/Data/ChiP-Seq-MouseESCs_2i/ESC_2i_ChiP_Seq_Sample_Table.csv"))
```

# 2 About this document

In this document, we describe how the golonska data matrices have been obtained. We downloaded the data from the SRA at the european nucleotide archive (ENA, accession PRJNA242892) and the peak lists in NARROWPEAK format from GEO (GSE56312).

The reads were aligned ot the mm9 reference genome using bowtie2 with default options. Then, filtering of unmapped, low mapping quality (< 10), duplicated and multi-mapping reads was performed Picard. Here, we use the resulting bam files as starting point.

Our sample table looks like this:

```
knitr::kable(sample_table)
```

| NCBI\_ID | SRA\_Exp\_ID | SRA\_Run\_ID | sample | SPP\_strand\_shift | ENA\_link | read\_length | bam | bam\_paths |
| --- | --- | --- | --- | --- | --- | --- | --- | --- |
| GSM1842747 | SRX1133072 | SRR2144628 | mES\_WCE | 150 | ftp://ftp.sra.ebi.ac.uk/vol1/fastq/SRR214/008/SRR2144628/SRR2144628.fastq.gz | 36 | SRR2144628\_WCE\_bowtie2\_mapped-only\_XS-filt\_no-dups.bam | /g/huber/users/klaus/Data/ChiP-Seq-MouseESCs\_2i/galaxy\_out/data/bam/mapped-only\_XS-filt\_nodups/SRR2144628\_WCE\_bowtie2\_mapped-only\_XS-filt\_no-dups.bam |
| GSM1842759 | SRX1133084 | SRR2144640 | mES\_H3K4me3\_Serum\_r1 | 265 | ftp://ftp.sra.ebi.ac.uk/vol1/fastq/SRR214/000/SRR2144640/SRR2144640.fastq.gz | 49 | SRR2144640\_H3K4me3\_Serum\_r1\_bowtie2\_mapped-only\_XS-filt\_no-dups.bam | /g/huber/users/klaus/Data/ChiP-Seq-MouseESCs\_2i/galaxy\_out/data/bam/mapped-only\_XS-filt\_nodups/SRR2144640\_H3K4me3\_Serum\_r1\_bowtie2\_mapped-only\_XS-filt\_no-dups.bam |
| GSM1842760 | SRX1133085 | SRR2144641 | mES\_H3K4me3\_Serum\_r2 | 260 | ftp://ftp.sra.ebi.ac.uk/vol1/fastq/SRR214/001/SRR2144641/SRR2144641.fastq.gz | 49 | SRR2144641\_H3K4me3\_Serum\_r2\_bowtie2\_mapped-only\_XS-filt\_no-dups.bam | /g/huber/users/klaus/Data/ChiP-Seq-MouseESCs\_2i/galaxy\_out/data/bam/mapped-only\_XS-filt\_nodups/SRR2144641\_H3K4me3\_Serum\_r2\_bowtie2\_mapped-only\_XS-filt\_no-dups.bam |
| GSM1842757 | SRX1133082 | SRR2144638 | ES\_H3K4me3\_24h\_2i\_r1 | 260 | ftp://ftp.sra.ebi.ac.uk/vol1/fastq/SRR214/008/SRR2144638/SRR2144638.fastq.gz | 49 | SRR2144638\_H3K4me3\_24h\_2i\_r1\_bowtie2\_mapped-only\_XS-filt\_no-dups.bam | /g/huber/users/klaus/Data/ChiP-Seq-MouseESCs\_2i/galaxy\_out/data/bam/mapped-only\_XS-filt\_nodups/SRR2144638\_H3K4me3\_24h\_2i\_r1\_bowtie2\_mapped-only\_XS-filt\_no-dups.bam |
| GSM1842762 | SRX1133087 | SRR2144643 | mES\_H3K4me3\_24h\_2i\_r2 | 260 | ftp://ftp.sra.ebi.ac.uk/vol1/fastq/SRR214/003/SRR2144643/SRR2144643.fastq.gz | 49 | SRR2144643\_H3K4me3\_24h\_2i\_r2\_bowtie2\_mapped-only\_XS-filt\_no-dups.bam | /g/huber/users/klaus/Data/ChiP-Seq-MouseESCs\_2i/galaxy\_out/data/bam/mapped-only\_XS-filt\_nodups/SRR2144643\_H3K4me3\_24h\_2i\_r2\_bowtie2\_mapped-only\_XS-filt\_no-dups.bam |

# 3 Import peak lists from paper and annotate nearest TSS

Here we import the peaklists from the paper for the serum and 24h\_2i condtion for the H3Kme3 histone modification. Then we use the function `toGRanges` from *ChIPpeakAnno* to import them as GRanges objects. We then join them into a common peaklist.

We then annotate them to the TSS that are close to them. We will later use only those genes to produce the profiles.

```
peak_files <- list.files(file.path("/g/huber/users/klaus/Data/ChiP-Seq-MouseESCs_2i/peak_lists_serum_2i_H3K4me3"), pattern = "*.NARROWPEAK", full.names = TRUE)

serum_idx <- str_detect(peak_files, "Serum")

peaks_serum <- toGRanges(peak_files[serum_idx], 
                           format="narrowPeak", header=FALSE)
```

```
   Warning in formatStrand(strand): All the characters for strand, 
               other than '1', '-1', '+', '-' and '*', 
               will be converted into '*'.
```

```
peaks_24h_2i <- toGRanges(peak_files[!serum_idx], 
                           format="narrowPeak", header=FALSE)
```

```
   Warning in formatStrand(strand): All the characters for strand, 
               other than '1', '-1', '+', '-' and '*', 
               will be converted into '*'.
```

```
all_peaks <- union(peaks_serum, peaks_24h_2i)


all_peaks_annotated <- annotatePeakInBatch(all_peaks, 
                                           AnnotationData = mus_genes)
```

# 4 Get the TSS

We now get the genes to which the peaks have been associated too, i.e. to which they are close. We then use the function `promotors` from `Biocpkg("Granges")` to get the TSS positions and add the entrez IDs as metadata.

```
genes_to_sel <- names(mus_genes) %in% mcols(all_peaks_annotated)$feature 
TSS_selected <- promoters(subset(mus_genes, genes_to_sel), 
                        upstream = 0, downstream = 1)

mcols(TSS_selected) <- DataFrame(entrez_id = names(TSS_selected))

#mus_tss <- transcriptsBy(mus_trans_ome, by = "exon")
```

# 5 Get the count tables for the samples

We now get the count tables for the samples using *soGGi*’s function `regionPlot`. We first import the data and then extract the count matrices. Since we have only one Input sample, we use this as Input data for all 4 experimental samples.

```
.soGGi_import_func <- function(bam, fragment_length, sample_id){
    
regionPlot(bam, TSS_selected, style="point", 
           distanceUp = 1000, distanceDown = 1500,
           format="bam", paired = FALSE, 
           removeDup=FALSE, FragmentLength = fragment_length,
           samplename = sample_id, nOfWindows = 2501)


}

## create list of args to iterate over

args_counting <- map(dplyr::select(sample_table, bam_paths, 
                                  SPP_strand_shift, sample), as.list)
names(args_counting) <- c("bam", "fragment_length", "sample_id")

count_tables <- pmap(args_counting, .soGGi_import_func)
```

```
   Reading Bam header information...
```

```
   ..Done
```

```
   Filtering regions which extend outside of genome boundaries...
```

```
   ..Done
```

```
   Filtered 0 of 13876 regions
```

```
   Splitting regions by Watson and Crick strand..
```

```
   ..Done
   ..Done
```

```
   Found 6990 Watson strand regions
```

```
   Found 6886 Crick strand regions
```

```
   Extending regions..
```

```
   ...done
```

```
   Reading tags from /g/huber/users/klaus/Data/ChiP-Seq-MouseESCs_2i/galaxy_out/data/bam/mapped-
   only_XS-filt_nodups/SRR2144628_WCE_bowtie2_mapped-only_XS-filt_no-dups.bam
```

```
   ..Done.
   Read in 1002095 reads
```

```
   Extending reads to fragmentlength of 150
```

```
   ..done
```

```
   Calculating coverage..
```

```
   ..done
```

```
   Calculating coverage across regions
   Calculating per contig.
```

```
   contig: 1
```

```
   contig: 2
```

```
   contig: 3
```

```
   contig: 4
```

```
   contig: 5
```

```
   contig: 6
```

```
   contig: 7
```

```
   contig: 8
```

```
   contig: 9
```

```
   contig: 10
```

```
   contig: 11
```

```
   contig: 12
```

```
   contig: 13
```

```
   contig: 14
```

```
   contig: 15
```

```
   contig: 16
```

```
   contig: 17
```

```
   contig: 18
```

```
   contig: 19
```

```
   contig: 20
```

```
   contig: 21
```

```
   contig: 22
```

```
   contig: 23
```

```
   contig: 24
```

```
   contig: 25
```

```
   contig: 26
```

```
   contig: 27
```

```
   contig: 28
```

```
   contig: 29
```

```
   contig: 30
```

```
   contig: 31
```

```
   contig: 32
```

```
   contig: 33
```

```
   contig: 34
```

```
   contig: 35
```

```
   Creating ChIPprofile.
```

```
   Reading Bam header information...
```

```
   ..Done
```

```
   Filtering regions which extend outside of genome boundaries...
```

```
   ..Done
```

```
   Filtered 0 of 13876 regions
```

```
   Splitting regions by Watson and Crick strand..
```

```
   ..Done
   ..Done
```

```
   Found 6990 Watson strand regions
```

```
   Found 6886 Crick strand regions
```

```
   Extending regions..
```

```
   ...done
```

```
   Reading tags from /g/huber/users/klaus/Data/ChiP-Seq-MouseESCs_2i/galaxy_out/data/bam/mapped-
   only_XS-filt_nodups/SRR2144640_H3K4me3_Serum_r1_bowtie2_mapped-only_XS-filt_no-dups.bam
```

```
   ..Done.
   Read in 2073462 reads
```

```
   Extending reads to fragmentlength of 265
```

```
   ..done
```

```
   Calculating coverage..
```

```
   ..done
```

```
   Calculating coverage across regions
   Calculating per contig.
```

```
   contig: 1
```

```
   contig: 2
```

```
   contig: 3
```

```
   contig: 4
```

```
   contig: 5
```

```
   contig: 6
```

```
   contig: 7
```

```
   contig: 8
```

```
   contig: 9
```

```
   contig: 10
```

```
   contig: 11
```

```
   contig: 12
```

```
   contig: 13
```

```
   contig: 14
```

```
   contig: 15
```

```
   contig: 16
```

```
   contig: 17
```

```
   contig: 18
```

```
   contig: 19
```

```
   contig: 20
```

```
   contig: 21
```

```
   contig: 22
```

```
   contig: 23
```

```
   contig: 24
```

```
   contig: 25
```

```
   contig: 26
```

```
   contig: 27
```

```
   contig: 28
```

```
   contig: 29
```

```
   contig: 30
```

```
   contig: 31
```

```
   contig: 32
```

```
   contig: 33
```

```
   contig: 34
```

```
   contig: 35
```

```
   Creating ChIPprofile.
```

```
   Reading Bam header information...
```

```
   ..Done
```

```
   Filtering regions which extend outside of genome boundaries...
```

```
   ..Done
```

```
   Filtered 0 of 13876 regions
```

```
   Splitting regions by Watson and Crick strand..
```

```
   ..Done
   ..Done
```

```
   Found 6990 Watson strand regions
```

```
   Found 6886 Crick strand regions
```

```
   Extending regions..
```

```
   ...done
```

```
   Reading tags from /g/huber/users/klaus/Data/ChiP-Seq-MouseESCs_2i/galaxy_out/data/bam/mapped-
   only_XS-filt_nodups/SRR2144641_H3K4me3_Serum_r2_bowtie2_mapped-only_XS-filt_no-dups.bam
```

```
   ..Done.
   Read in 3777014 reads
```

```
   Extending reads to fragmentlength of 260
```

```
   ..done
```

```
   Calculating coverage..
```

```
   ..done
```

```
   Calculating coverage across regions
   Calculating per contig.
```

```
   contig: 1
```

```
   contig: 2
```

```
   contig: 3
```

```
   contig: 4
```

```
   contig: 5
```

```
   contig: 6
```

```
   contig: 7
```

```
   contig: 8
```

```
   contig: 9
```

```
   contig: 10
```

```
   contig: 11
```

```
   contig: 12
```

```
   contig: 13
```

```
   contig: 14
```

```
   contig: 15
```

```
   contig: 16
```

```
   contig: 17
```

```
   contig: 18
```

```
   contig: 19
```

```
   contig: 20
```

```
   contig: 21
```

```
   contig: 22
```

```
   contig: 23
```

```
   contig: 24
```

```
   contig: 25
```

```
   contig: 26
```

```
   contig: 27
```

```
   contig: 28
```

```
   contig: 29
```

```
   contig: 30
```

```
   contig: 31
```

```
   contig: 32
```

```
   contig: 33
```

```
   contig: 34
```

```
   contig: 35
```

```
   Creating ChIPprofile.
```

```
   Reading Bam header information...
```

```
   ..Done
```

```
   Filtering regions which extend outside of genome boundaries...
```

```
   ..Done
```

```
   Filtered 0 of 13876 regions
```

```
   Splitting regions by Watson and Crick strand..
```

```
   ..Done
   ..Done
```

```
   Found 6990 Watson strand regions
```

```
   Found 6886 Crick strand regions
```

```
   Extending regions..
```

```
   ...done
```

```
   Reading tags from /g/huber/users/klaus/Data/ChiP-Seq-MouseESCs_2i/galaxy_out/data/bam/mapped-
   only_XS-filt_nodups/SRR2144638_H3K4me3_24h_2i_r1_bowtie2_mapped-only_XS-filt_no-dups.bam
```

```
   ..Done.
   Read in 1365978 reads
```

```
   Extending reads to fragmentlength of 260
```

```
   ..done
```

```
   Calculating coverage..
```

```
   ..done
```

```
   Calculating coverage across regions
   Calculating per contig.
```

```
   contig: 1
```

```
   contig: 2
```

```
   contig: 3
```

```
   contig: 4
```

```
   contig: 5
```

```
   contig: 6
```

```
   contig: 7
```

```
   contig: 8
```

```
   contig: 9
```

```
   contig: 10
```

```
   contig: 11
```

```
   contig: 12
```

```
   contig: 13
```

```
   contig: 14
```

```
   contig: 15
```

```
   contig: 16
```

```
   contig: 17
```

```
   contig: 18
```

```
   contig: 19
```

```
   contig: 20
```

```
   contig: 21
```

```
   contig: 22
```

```
   contig: 23
```

```
   contig: 24
```

```
   contig: 25
```

```
   contig: 26
```

```
   contig: 27
```

```
   contig: 28
```

```
   contig: 29
```

```
   contig: 30
```

```
   contig: 31
```

```
   contig: 32
```

```
   contig: 33
```

```
   contig: 34
```

```
   contig: 35
```

```
   Creating ChIPprofile.
```

```
   Reading Bam header information...
```

```
   ..Done
```

```
   Filtering regions which extend outside of genome boundaries...
```

```
   ..Done
```

```
   Filtered 0 of 13876 regions
```

```
   Splitting regions by Watson and Crick strand..
```

```
   ..Done
   ..Done
```

```
   Found 6990 Watson strand regions
```

```
   Found 6886 Crick strand regions
```

```
   Extending regions..
```

```
   ...done
```

```
   Reading tags from /g/huber/users/klaus/Data/ChiP-Seq-MouseESCs_2i/galaxy_out/data/bam/mapped-
   only_XS-filt_nodups/SRR2144643_H3K4me3_24h_2i_r2_bowtie2_mapped-only_XS-filt_no-dups.bam
```

```
   ..Done.
   Read in 1056552 reads
```

```
   Extending reads to fragmentlength of 260
```

```
   ..done
```

```
   Calculating coverage..
```

```
   ..done
```

```
   Calculating coverage across regions
   Calculating per contig.
```

```
   contig: 1
```

```
   contig: 2
```

```
   contig: 3
```

```
   contig: 4
```

```
   contig: 5
```

```
   contig: 6
```

```
   contig: 7
```

```
   contig: 8
```

```
   contig: 9
```

```
   contig: 10
```

```
   contig: 11
```

```
   contig: 12
```

```
   contig: 13
```

```
   contig: 14
```

```
   contig: 15
```

```
   contig: 16
```

```
   contig: 17
```

```
   contig: 18
```

```
   contig: 19
```

```
   contig: 20
```

```
   contig: 21
```

```
   contig: 22
```

```
   contig: 23
```

```
   contig: 24
```

```
   contig: 25
```

```
   contig: 26
```

```
   contig: 27
```

```
   contig: 28
```

```
   contig: 29
```

```
   contig: 30
```

```
   contig: 31
```

```
   contig: 32
```

```
   contig: 33
```

```
   contig: 34
```

```
   contig: 35
```

```
   Creating ChIPprofile.
```

```
names(count_tables) <- args_counting$sample_id


# function to extract the data matrix and summarize it across positions

.get_data_matrix <- function(count_tables){
  
  ret <-  map(count_tables, function(X){
  
    mat <- assay(X)
    summarizeCountsPerPosition(mat)
    
  })
  
  ret <- do.call(cbind, ret)
  
}

mat <- .get_data_matrix(count_tables)


input_galonska <- cbind(mat[, "mES_WCE"], mat[, "mES_WCE"], mat[, "mES_WCE"], mat[, "mES_WCE"] )

chip_galonska <- mat[,2:5]
```

```
sample_table_galonska <- data.frame(sampleID = sample_table$sample[2:5],
                                    fragment_length = sample_table$SPP_strand_shift[2:5],
                                    input_fragment_length = rep(sample_table$SPP_strand_shift[1],4),
                                    input = rep(sample_table$sample[1], 4),
                                    upstream = rep(1000, 4),
                                    downstream = rep(1500, 4),
                                    condition = c("Serum", "Serum",
                                                  "24h_2i", "24h_2i"))
```

```
save(input_galonska, chip_galonska, sample_table_galonska,
     file = "galonskaData.rda", compress = "xz")
```

```
TSS_galonska <- TSS_selected

save(TSS_galonska, file = "TSS_galonska.rda", compress = "xz")
```

```
save(input_galonska, file = "input_galonska.rda", compress = "xz")
save(chip_galonska, file = "chip_galonska.rda", compress = "xz")
save(sample_table_galonska, file = "sample_table_galonska.rda", compress = "xz")
```

# 6 Output of sessionInfo()

```
sessionInfo()
```

```
   R version 3.2.2 (2015-08-14)
   Platform: x86_64-pc-linux-gnu (64-bit)
   Running under: CentOS release 6.5 (Final)
   
   locale:
    [1] LC_CTYPE=en_US.UTF-8       LC_NUMERIC=C              
    [3] LC_TIME=en_US.UTF-8        LC_COLLATE=en_US.UTF-8    
    [5] LC_MONETARY=en_US.UTF-8    LC_MESSAGES=en_US.UTF-8   
    [7] LC_PAPER=en_US.UTF-8       LC_NAME=C                 
    [9] LC_ADDRESS=C               LC_TELEPHONE=C            
   [11] LC_MEASUREMENT=en_US.UTF-8 LC_IDENTIFICATION=C       
   
   attached base packages:
    [1] grid      stats4    parallel  stats     graphics  grDevices utils    
    [8] datasets  methods   base     
   
   other attached packages:
    [1] MSnbase_1.18.1                         
    [2] ProtGenerics_1.2.1                     
    [3] BiocParallel_1.4.3                     
    [4] mzR_2.4.0                              
    [5] GenomicAlignments_1.6.3                
    [6] Rsamtools_1.22.0                       
    [7] soGGi_1.2.1                            
    [8] rtracklayer_1.30.2                     
    [9] readr_0.2.2                            
   [10] ChIPpeakAnno_3.4.6                     
   [11] RSQLite_1.0.0                          
   [12] DBI_0.3.1                              
   [13] Biostrings_2.38.4                      
   [14] XVector_0.10.0                         
   [15] TxDb.Mmusculus.UCSC.mm9.knownGene_3.2.2
   [16] GenomicFeatures_1.22.13                
   [17] AnnotationDbi_1.32.3                   
   [18] xtable_1.8-2                           
   [19] gplots_2.17.0                          
   [20] purrr_0.2.1                            
   [21] VennDiagram_1.6.16                     
   [22] futile.logger_1.4.1                    
   [23] smoothmest_0.1-2                       
   [24] MASS_7.3-45                            
   [25] reshape2_1.4.1                         
   [26] fdrtool_1.2.15                         
   [27] edgeR_3.12.0                           
   [28] limma_3.26.8                           
   [29] DESeq2_1.10.1                          
   [30] RcppArmadillo_0.6.500.4.0              
   [31] Rcpp_0.12.3                            
   [32] csaw_1.4.1                             
   [33] SummarizedExperiment_1.0.2             
   [34] Biobase_2.30.0                         
   [35] GenomicRanges_1.22.4                   
   [36] GenomeInfoDb_1.6.3                     
   [37] IRanges_2.4.8                          
   [38] S4Vectors_0.8.11                       
   [39] BiocGenerics_0.16.1                    
   [40] tidyr_0.4.1                            
   [41] biomaRt_2.26.1                         
   [42] devtools_1.10.0                        
   [43] openxlsx_3.0.0                         
   [44] pheatmap_1.0.8                         
   [45] stringr_1.0.0                          
   [46] RColorBrewer_1.1-2                     
   [47] dplyr_0.4.3                            
   [48] plyr_1.8.3                             
   [49] ggplot2_2.0.0                          
   [50] DChIPRep_1.0.4                         
   [51] knitr_1.12.3                           
   [52] BiocStyle_1.8.0                        
   
   loaded via a namespace (and not attached):
    [1] colorspace_1.2-6             hwriter_1.3.2               
    [3] affyio_1.40.0                interactiveDisplayBase_1.8.0
    [5] codetools_0.2-14             splines_3.2.2               
    [7] doParallel_1.0.10            impute_1.44.0               
    [9] geneplotter_1.48.0           Formula_1.2-1               
   [11] annotate_1.48.0              vsn_3.38.0                  
   [13] cluster_2.0.3                GO.db_3.2.2                 
   [15] graph_1.48.0                 shiny_0.13.1                
   [17] httr_1.1.0                   lazyeval_0.1.10             
   [19] assertthat_0.1               formatR_1.2.1               
   [21] acepack_1.3-3.3              htmltools_0.3               
   [23] tools_3.2.2                  affy_1.48.0                 
   [25] gtable_0.2.0                 ShortRead_1.28.0            
   [27] MALDIquant_1.14              multtest_2.26.0             
   [29] gdata_2.17.0                 preprocessCore_1.32.0       
   [31] iterators_1.0.8              mime_0.4                    
   [33] ensembldb_1.2.2              gtools_3.5.0                
   [35] XML_3.98-1.3                 AnnotationHub_2.2.3         
   [37] zlibbioc_1.16.0              scales_0.4.0                
   [39] BSgenome_1.38.0              BiocInstaller_1.20.1        
   [41] pcaMethods_1.60.0            RBGL_1.46.0                 
   [43] lambda.r_1.1.7               yaml_2.1.13                 
   [45] memoise_1.0.0                gridExtra_2.2.1             
   [47] rpart_4.1-10                 latticeExtra_0.6-28         
   [49] stringi_1.0-1                genefilter_1.52.1           
   [51] foreach_1.4.3                caTools_1.17.1              
   [53] chipseq_1.20.0               bitops_1.0-6                
   [55] matrixStats_0.50.1           mzID_1.8.0                  
   [57] evaluate_0.8                 lattice_0.20-33             
   [59] magrittr_1.5                 R6_2.1.2                    
   [61] Hmisc_3.17-2                 foreign_0.8-66              
   [63] survival_2.38-3              RCurl_1.95-4.7              
   [65] nnet_7.3-12                  futile.options_1.0.0        
   [67] KernSmooth_2.23-15           rmarkdown_0.9.5             
   [69] locfit_1.5-9.1               digest_0.6.9                
   [71] httpuv_1.3.3                 regioneR_1.2.3              
   [73] munsell_0.4.3
```
